# Studies on the interactions of Ag(i) with DNA and their implication on the DNA-templated synthesis of silver nanoclusters and on the interaction with complementary DNA and RNA sequences†

**DOI:** 10.1039/d1ra00194a

**Published:** 2021-03-01

**Authors:** Alejandra de la Hoz, Alba Navarro, Anna Aviñó, Ramon Eritja, Raimundo Gargallo

**Affiliations:** Dept. of Chemical Engineering and Analytical Chemistry, University of Barcelona Marti i Franquès 1 E-08028 Barcelona Spain raimon_gargallo@ub.edu; Institute for Advanced Chemistry of Catalonia (IQAC-CSIC), CIBER-BBN Jordi Girona 18-26 E-08034 Barcelona Spain

## Abstract

Silver nanoclusters (AgNCs) prepared by the reduction of silver ions in the presence of DNA oligonucleotides have attracted great interest as potential diagnostic tools for their tunable and high fluorescent properties. In this work, three DNA sequences that consist of a 12-nucleotide long probe sequence at the 5′-end linked to the complementary sequence to three miRNAs are studied. First, the interaction of these sequences with Ag(i) was characterized by means of circular dichroism spectroscopy. By applying multivariate methods to the analysis of spectroscopic data, two complexes with different Ag(i) : DNA ratios were resolved. Secondly, the impact of several experimental variables, such as temperature, borohydride concentration and reaction time, on the formation of AgNCs templated by these three sequences was studied. Finally, the fluorescence properties of the duplexes formed by DNA probes with complementary DNA or miRNA sequences were studied. The results presented here highlight the role of the secondary structure adopted by the DNA probe on the fluorescence properties of DNA-stabilized AgNCs which, in turn, affect the development of methods for miRNA detection.

## Abstract 2

Variables affecting the fluorescent properties of DNA-stabilized silver nanoclusters are studied. The secondary structure of the AgNC-stabilizing DNA sequence dramatically affects the analytical signal behind the hybridization reaction.

## Graphical abstract

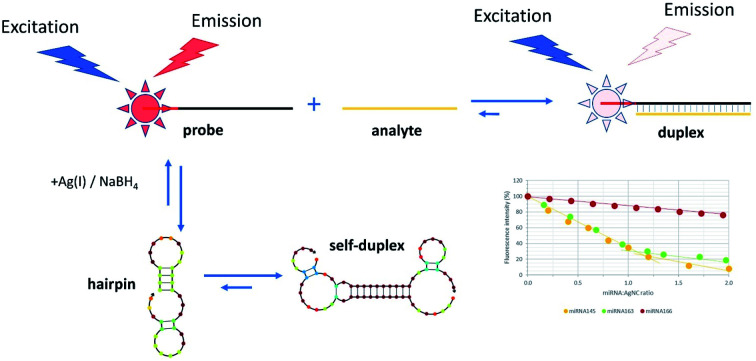

## Introduction

Metal nanoclusters (NCs) are one of the most important contributions to nanotechnology to nanomedicine due to their excellent physical and chemical properties.^1^ NCs are tiny (less than 2 nm) groups of a few metal atoms that have quantized molecule-like orbitals, enabling the existence of spectroscopic phenomena, such as fluorescence.^2,3^ Extensive and critical reviews on this topic have been published.^4–6^ Specifically, silver nanoclusters (AgNCs) prepared by the reduction of silver ions in the presence of short DNA oligonucleotides have attracted great interest as potential diagnostic tools for their tunable and highly fluorescent properties.^7^ Recently, the use of RNA nanorings to encapsulate AgNCs has also been described.^8^ Although studies have been conducted to try to correlate DNA sequences and lengths with the fluorescence properties of AgNCs,^9^ the significance of the secondary structures of the DNA templates on the emission properties of the resulting AgNCs is still a matter of research.^10–16^

miRNAs are small non-coding RNA molecules that play a key role in RNA silencing and post-transcriptional regulation of gene expression. Because of this, they are considered excellent biomarkers for a variety of pathological states such as cancer and neurodegenerative diseases, since they have been found to be overexpressed in affected cells. Therefore, their detection is important for the diagnosis of early and advanced states of these diseases and, consequently, there is a need for quantitative and specific methods to analyze miRNAs. Commonly used detection methods for miRNA detection have recently been reviewed.^17–20^ Currently, the most widely used methods for analyzing microRNAs are quantitative reverse transcription PCR (qRT-PCR), Northern blotting, *in situ* hybridization, microarrays and next-generation sequencing.^18^ Also, several strategies involving DNA-stabilized AgNCs for miRNA detection have been reviewed, including the generation of AgNCs and quenching of their fluorescent signal.

In preliminary work,^21^ the detection of miRNA160 was accomplished by hybridization of miRNA160 to a probe consisting of a DNA sequence built from the assembly of a 12-nucleotide long sequence (named 12red) and the complementary strand to miRNA160. This probe displayed strong emission from the AgNCs after addition of AgNO_3_ and reduction with NaBH_4_ (DNA/AgNO_3_/NaBH_4_ in a 1 : 17 : 17 ratio). The presence of miRNA160 was detected by hybridization with the complementary probe that provoked the inhibition of the fluorescence (“turn-off” strategy). Based on this work, other approaches for the detection of miRNAs were proposed. Xia *et al.* described a hairpin DNA with 5′ and 3′ overhangs for the detection of miRNAs.^22^ The use of DNA/RNA chimera templates, which show more intense red fluorescence than their DNA counterparts, has also been proposed.^23^ Recently, the development of a dual emitter from red to green in the absence and presence of target miRNA, respectively, has been described.^24^

In an effort to find an analytical method to quantify simultaneously several miRNAs based on the variation in fluorescence by hybridization with DNA-stabilized AgNC probes, we studied the key factors that may influence the precise detection of these molecules. First, we focused our attention on the interaction of Ag(i) with the selected probes, as well as the initial optimization of the AgNC synthesis. Then we studied the fluorescence behavior for the quantification of several miRNAs by hybridization with the corresponding DNA-AgNC probes.

The DNA sequences used in this work are given in Table 1. The design of these sequences follows previously described protocols in which there is a 12 nucleotide cytosine-rich sequence that directs the synthesis of a specific AgNC (reporter DNA sequence) connected with a DNA sequence complementary to three miRNAs (miRNA145, miRNA163 and miRNA166). The selected 12-nucleotide reporter sequences have been described previously for the development of AgNCs showing different spectral properties.^25^ Here, we demonstrate that the structure of the resulting DNA probes is a critical factor in the biophysical properties of the AgNCs and it affects the potential applicability of the AgNCs for the detection of miRNAs.

**Table 1:** DNA sequences studied in this work. Highlighted nucleotides denote the sensing sequences, as well as the expected colors in the fluorescence spectra

Name	Sequence (5′ → 3′)	Length
12red	**CCT CCT TCC TCC**	12
12redComp145	**CCT CCT TCC TCC** AG GGA TTC CTG GGA AAA CTG GAC	35
DNA145	GT CCA GTT TTC CCA GGA ATC CCT	23
miRNA145	GU CCA GUU UUC CCA GGA AUC CCU	23
12yellow	**CCC TTA ATC CCC**	12
12yellowComp163	**CCC TTA ATC CCC** ATC GAA GTT CCA AGT CCT CTT CAA	36
DNA163	TTG AAG AGG ACT TGG AAC TTC GAT	24
miRNA163	UUG AAG AGG ACU UGG AAC UUC GAU	24
12IR	**CCC TAA CTC CCC**	12
12IRComp166	**CCC TAA CTC CCC** GGG GAA TGA AGC CTG GTC CGA	33
DNA166	TCG GAC CAG GCT TCA TTC CCC	21
miRNA166	UCG GAC CAG GCU UCA UUC CCC	21

## Experimental section

### Chemicals and DNA sequences

The DNA sequences (Table 1) were either purchased from Merck (Darmstadt, Germany) or synthesized on an Applied Biosystems 3400 DNA synthesizer using the 1 μM scale synthesis cycle. In this latter case, standard phosphoramidites were used and ammonia deprotection was performed overnight at 55 °C. The resulting products were purified using a Glen-Pak Purification Cartridge (Glen Research). The integrity of all DNA sequences was checked by means of MALDI-TOF mass spectrometry. DNA strand concentration was determined by absorbance measurements (260 nm) at 90 °C using the extinction coefficients calculated using the nearest-neighbor method as implemented on the OligoCalc webpage.^26^ Potassium phosphate buffer (pH 7.2), AgNO_3_ and NaBH_4_ were purchased from Merck (Darmstadt, Germany). MilliQ water was used in all experiments.

### Procedure for AgNC synthesis

Initially, DNA-stabilized AgNCs were synthesized using the procedures described in the literature.^27^ In an Eppendorf® vial containing phosphate buffer, an aliquot of the oligonucleotide stock solution was introduced. A certain volume of AgNO_3_ was then added and allowed to stand for 15 minutes in ice. Finally, freshly prepared NaBH_4_ was added and the solution was stirred vigorously for 1 minute. The final buffer concentration was 5 mM. The synthesized DNA-AgNCs were stored at 4 °C in the dark overnight before measurement. The AgNO_3_ stock solution was prepared by dissolving in a 10 mL volumetric flask the mass of AgNO_3_ required to obtain a 1 M concentration solution. A 1 × 10^−3^ M AgNO_3_ solution was obtained through a dilution cascade, and was stored at room temperature in an amber volumetric flask to prevent oxidation. The NaBH_4_ stock solution was prepared by dissolving the appropriate amount of solid NaBH_4_ in a 25 mL volumetric flask to obtain a 0.1 M solution. From this, a 1 × 10^−3^ M NaBH_4_ solution was obtained by dilution, which was kept on ice until use. The steps concerning the preparation and addition of borohydride were later modified, as described below.

### Instruments and apparatus

Absorbance spectra were recorded on an Agilent 8453 diode array spectrophotometer. Hellma quartz cells (10 mm path length, 400 and 1500 μL volume) were used. Circular dichroism (CD) spectra were recorded on a Jasco J-810 spectropolarimeter equipped with a Peltier accessory for temperature control. This instrument enabled the simultaneous acquisition of both CD and molecular absorption spectra. Hellma quartz cells (10 mm path length, 3000 μl volume) were used.

Melting experiments were monitored using Agilent 8453 and the Jasco J-810 instruments. The sample solution was transferred to a covered 10 mm-path-length cell and spectra were recorded at 2 °C intervals with a holding time of 3 minutes at each temperature, which yielded an average heating rate of approximately 0.6 °C min^−1^.

Fluorescence experiments were monitored using an AB2 Aminco-Bowman spectrofluorometer. For most of the measurements, excitation and emission slits were set to 4 nm, and the voltage of the photomultiplier was set to 800 V. A Hellma quartz cell (2 × 10 mm path length, and 400 μl volume) was used. For each measurement, two emission spectra were recorded, averaged, and later smoothed by applying a Savitzky–Golay filter (41 points, third-degree polynomial). The following spectral ranges are considered in the paper: green (501–565 nm), yellow (566–590 nm), orange (591–625 nm), and red (626–740 nm).

For SE-HPLC, the chromatographic system consisted of a Waters 2695 HPLC instrument equipped with a quaternary pump, a degasser, an autosampler, a photodiode-array detector with a 13 μL flow cell, and software for data acquisition and analysis. The chromatographic column used for separation at room temperature was a PSS Suprema Analytical Lineal S 100–100 000 Da (PSS Polymer Standards Service GmbH, Mainz, Germany). The composition of the mobile phase was 5 mM phosphate. The flow was set to 0.8 mL min^−1^. The injection volume was 15 μL. *T*_15_, *T*_20_, *T*_25_, *T*_20_ and *T*_45_ sequences were used as standards to construct a plot of the logarithm of the retention time (*t*_R_) *vs.* molecular weight. The *T*_15_ and *T*_45_ standards were injected twice to assess the reproducibility of the *t*_R_ values (±0.01 minutes). SE-HPLC profiles were normalized to equal length (Euclidean normalization) to eliminate potential variations in the DNA concentration of samples that could hinder the comparison of chromatograms. Normalization was carried out using eqn (1). The variable *d*_*i*_ indicates the value of absorbance at time *i*, and *n* is the total number of points in each chromatogram.1
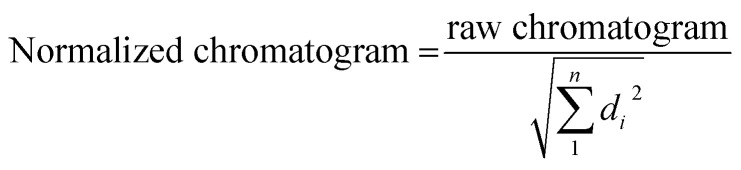


### Multivariate analysis

Multivariate analysis uses data recorded at more than one channel. In the case of molecular absorption or CD spectroscopies, a channel corresponds to a wavelength at which absorbance or ellipticity was measured during the considered experiment. Accordingly, either absorbance or CD spectra recorded during melting or titration of DNA with Ag(i) were arranged in a matrix **D**, the dimensions of which were *m* spectra × *n* channels. For instrumental techniques that provide a linear signal *vs.* concentration response, it is possible to decompose this matrix **D** according to the equation:

2 **D** = **C** × **S** + **E**

**C** is a matrix that contains the distribution diagram for all the components present during the titration. The dimensions of matrix **C** are *m* × nc, where nc is the proposed number of components. **S** is the matrix that contains the absorbance or CD spectra of each of these components, also called pure spectra. The dimensions of **S** are nc × *n*. Finally, **E** is the matrix (*m* × *n*) of experimental data not explained by the multiplication of **C** by **S**. For the appropriate number of components, data in **E** should be randomly distributed.

In this work, decomposition of matrix **D** has been done by using the Multivariate Curve Resolution based on an Alternating Least Squares (MCR-ALS) procedure. Briefly, the decomposition of **D** according to eqn (2) is accomplished by an iterative mode at the end of which matrices **C** and **S** are calculated. The calculation implies the application of several constraints to the values contained in matrix **C**, like the closure concentration (*i.e.*, the sum of concentrations of all components is forced to be constant) or non-negativity of concentration values. A fuller description of this procedure, as well as its many applications in Chemistry and Biophysics, may be found in previous work.^28–30^

## Results

### Characterization of DNA sequences

Firstly, a prediction of the intermolecular and intramolecular folding of all sequences was done by *in silico* calculations using either the *mfold*^31^ or OligoAnalyzer tools^32^ (Fig. S1†). The shorter sequences were unable to fold intramolecularly into a hairpin. However, 12yellow and 12IR may produce self-duplexes in minor concentrations under these conditions, with the one formed by 12yellow being slightly more stable (−4.8 kcal mol^−1^) than that formed by 12IR (−1.0 kcal mol^−1^). On the other hand, longer sequences were predicted to fold into intramolecular hairpins and self-duplexes. 12redComp145 is a sequence that is able to fold into a hairpin with a lower change in Gibbs free energy at 15 °C (−5.4 kcal mol^−1^), whereas the other two show similar stabilities (−2.3 and −2.4 kcal mol^−1^). The 12IRComp166 sequence is one that can fold into a self-duplex with a more negative Gibbs free energy (−25.2 kcal mol^−1^ at 15 °C). Overall, *in silico* calculations predicted that 12redComp145 and 12yellowComp163 would be mainly present as hairpins, whereas 12IRComp166 would fold into a self-duplex.

**Fig. 1:**
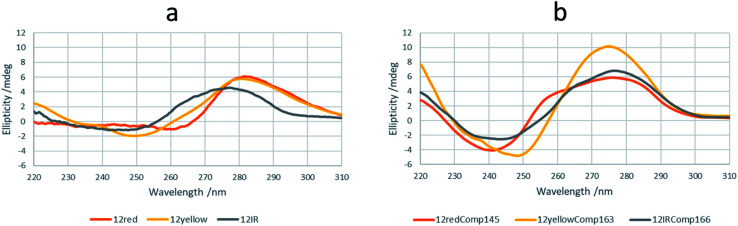
CD spectra of short (a) and long (b) sequences. The experimental conditions of the measurements were 2 μM DNA, 5 mM phosphate buffer, pH 7.1, 15 °C. Other instrumental conditions were as detailed in the text.

CD spectra of all sequences were measured at pH 7.1 and 15 °C (Fig. 1a). The CD spectrum of 12IR shows a positive band around 275 nm, which is indicative of an unfolded strand. The CD spectrum of 12yellow shows a positive band around 280 nm, a small negative band around 250 nm and a crossover around 260 nm, indicative of weak folding into a B-DNA conformation. Finally, the CD spectrum of 12red shows small negative and positive bands around 260 and 282 nm, respectively, and a crossover around 267 nm. These spectral features were slightly different from those observed for 12yellow, suggesting a different structure. To check this point, CD spectra were also measured at pH 5 (Fig. S2a†). The spectral characteristics (positive bands at 220 and 285, and a negative band at 260 nm) agreed with those of the i-motif structure^33^ (Fig. S2b†).

Finally, CD spectra of the probe sequences showed intense positive bands around 275 and negative bands around 240–250 nm, indicative of folding into a B-DNA conformation. Interestingly, the spectra of 12redComp145 and 12IRComp166 also show shoulders around 255 and 265 nm, respectively (Fig. 1b).

**Fig. 2:**
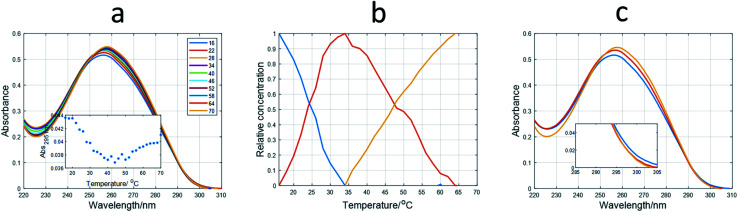
Multivariate analysis of spectra recorded during the melting experiment of 12IRComp166. (a) Experimental absorbance spectra. Inset shows the absorbance at 295 nm *vs. T*. (b) Calculated distribution diagram (matrix **C** in eqn (2), and (c) pure spectra (matrix **S** in eqn (2)) by means of MCR-ALS. The inset in (c) highlights the absorbance region in pure spectra around 300 nm.

Spectroscopically monitored melting experiments were done to characterize the studied sequences. For probe sequences, both absorbance and ellipticity traces showed smooth and broad unfolding processes. The whole set of spectra measured during the melting were analyzed by means of a multivariate data analysis method based on soft modeling.^28^ The main advantage of this procedure over univariate analysis is the use of information provided by several channels (wavelengths), which could help to uncover the presence of intermediate species. The method used in this work, MCR-ALS, does not need the proposal of any model concerning the nature (stoichiometry, folding) of the proposed species. For 12IRComp166, the absorbance trace at 295 nm suggested the presence of more than one unfolding process (Fig. 2a, inset). The analysis of the set of absorbance spectra with MCR-ALS showed the existence of three spectroscopically active components, which could be related to different DNA conformations. Accordingly, the distribution diagram showed two transitions centered around 23 and 47 °C, respectively (Fig. 2b). By using Nupack software^34^ the *T*_m_ value for the unfolding of the hairpin was calculated (45 °C), in agreement with the experimental observation. The transition at lower temperatures would be then related to the equilibrium between self-duplex and hairpin. The potential formation of the i-motif structure by the cytosine-rich fragment was ruled out from the absence of any clear CD signal at 288 nm in spectra recorded during an additional melting experiment (Fig. S3†). Finally, the spectral features observed in the experimental absorbance spectra were well explained by the calculated pure spectra (Fig. 2c), such as the hypochromicity observed at low temperatures.

For 12redComp145 and 12yellowComp163, the results showed a two-step unfolding process (see Fig. S4†). The smooth and small transition in these two cases agreed with the small degree of folding predicted by *in silico* methods.

### Binding of Ag(i) to DNA sequences

**Fig. 3:**
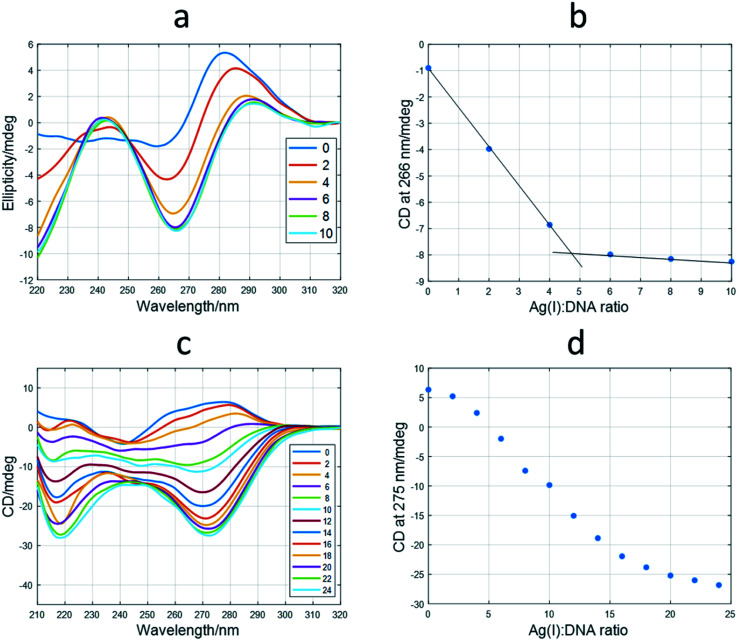
Titrations of 12red and 12redComp145 with Ag(i). (a) CD spectra recorded during the titration of 12red. The legend shows the Ag(i) : DNA ratio at which CD spectra were measured. (b) Variation of CD signal at 266 nm *vs.* Ag(i) : DNA ratio for 12red. (c) CD spectra recorded during the titration of 12redComp145. (d) Variation of CD signal at 275 nm *vs.* Ag(i) : DNA ratio for 12redComp145. The experimental conditions were 5 mM phosphate buffer, pH 7.1, 15 °C, DNA concentration 2 μM.

First, the interaction of the shortest sequences with Ag(i) was studied by means of CD spectroscopy. In the case of the 12red sequence, the addition of Ag(i) produced dramatic changes (Fig. 3a) on the initial unfolded structure. The spectrum at a ratio of 10 : 1 Ag(i) : DNA showed positive signals around 245 and 290 nm and a negative band around 265 nm. The position of the CD bands is similar to those corresponding to canonical i-motif structures. Hence, the folded structure adopted by 12red in the presence of Ag(i) would be similar to this structure, being the building block of the C·Ag(i)·C triplet, in a way that resembles the hydrogen bonding pattern present in the canonical i-motif structure, as suggested by other authors.^35,36^ However, this could not be the general situation for Ag(i)-induced folding of C-rich sequences. In this sense, a recent structural study based on X-ray diffraction on the dimer former by the A_2_C_4_ oligonucleotide showed that this sequence, which binds up to 8 Ag atoms, adopts an intermediate structure between A- and B-DNA structures, rather than that of an i-motif.^37^ In recent work, Swasey and Gwinn proposed the formation of duplex DNA structures stabilized by C–Ag(i)–C triplets.^38,39^ The shape of the CD spectra shown in these works was very similar to that shown in Fig. 3a, the main difference being the lack of the positive band at around 290 nm.

From the ellipticity trace at 266 nm, it could be deduced that the conformational change induced by Ag(i) binding the 12red sequence was finished at an approximate ratio of Ag(i) : DNA of 5 : 1 (Fig. 3b). Similar titrations were carried out for 12yellow and 12IR (Fig. S5†). These sequences showed spectral variations like those observed for 12red. Hence, it was deduced that the final structure adopted by all short oligonucleotides was similar. These short oligonucleotides were also able to accommodate up to five and six Ag(i) ions, respectively. In the case of 12IR, these results agree with those previously reported by Petty *et al.,* who determined a Ag(i) : DNA stoichiometry equal to 5.9 ± 0.5.^40^

As it would be expected, probe sequences showed a more complex behavior than that observed for short sequences. For example, CD spectra measured during the titration of 12redComp145 with Ag(i) are given in Fig. 3c. When comparing these spectra with those measured during the titration of 12red (Fig. 3a), it may be observed that positive bands around 240 and 285 nm have disappeared, whereas the negative band has shifted to approximately 275 nm. Moreover, the plot of ellipticity *vs.* Ag(i) : DNA ratio at 275 nm (Fig. 3d) showed the potential existence of intermediate species. Similar results were obtained for 12yellowComp163 and 12IRComp166 (Fig. S6†).

To gain insight into these processes, the whole set of spectra measured during the titrations of long sequences with Ag(i) were analyzed by means of a multivariate data analysis method based on soft modeling.

**Fig. 4:**
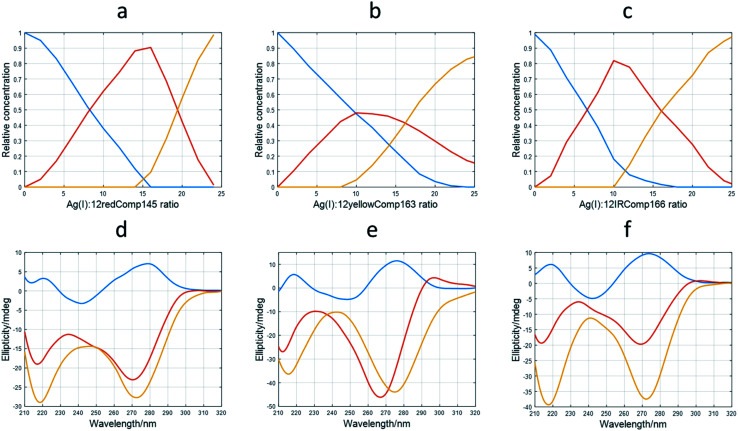
Results of multivariate analysis of CD-monitored titrations of long sequences with Ag(i) according to eqn (2). Calculated distribution diagrams for 12redComp145 (a), 12yellowComp163 (b) and 12IRComp166 (c). Calculated pure CD spectra for 12redComp145 (d), 12yellowComp163 (e) and 12IRComp166 (f). In all cases, profiles colored in blue correspond to free unbounded DNA. The profiles colored in red or orange correspond to different DNA : Ag(i) species.

The application of this procedure allowed the determination of the number of components (nc) present during the titration of DNA with Ag(i), as well as the corresponding distribution diagram (in this case, the plot of concentration of DNA species *vs.* Ag(i) : DNA ratio) and pure spectra for each of the *nc* components. Fig. 4a and d show the calculated profiles for 12redComp145. In this case, three components were proposed, and the corresponding figures of merit are given in Fig. S7.† The component shown in blue corresponds to unbound 12redComp145, which should be a hairpin, according to the *in silico* calculations. Upon addition of Ag(i), two successive complexes were resolved, reaching their maximal concentrations at stoichiometries around 14 : 1 and 22 : 1 for Ag(i) : DNA. This latter value should be considered critically because of the presence of underlying ambiguities inherent to soft modeling methods. Hence, the number of Ag(i) ions bound to the last complex, as well as the magnitude of the corresponding pure CD spectrum, are somewhat uncertain. The calculated pure spectra showed that the overall conformation of the two complexes was similar but differed in the number of silver ions in their complexes.

The analysis of 12yellowComp163 revealed the formation of two complexes with maximal formation at Ag(i) : DNA ratios equal to 10 : 1 and 25 : 1, respectively. As in the previous case, both the stoichiometry of the major complex at high Ag(i) : DNA ratios and the magnitude of its resolved pure CD spectrum are rather uncertain. Finally, the analysis of 12IRComp166 also revealed the formation of two complexes with Ag(i):ratios of approximately 10 : 1 and 22 : 1.

### Procedure for AgNC synthesis

AgNCs are usually prepared from the reduction of DNA : Ag(i) mixtures with sodium borohydride.^41^ Frequently, the procedures for such a synthesis that may be found in the literature mention the use of “fresh borohydride solution” and an aging period following the addition to the DNA : Ag(i) mixture that may last from 15 minutes^42^ or 1 hour^11,43^ at room temperature, to even overnight at 4 °C.^44^

**Fig. 5:**
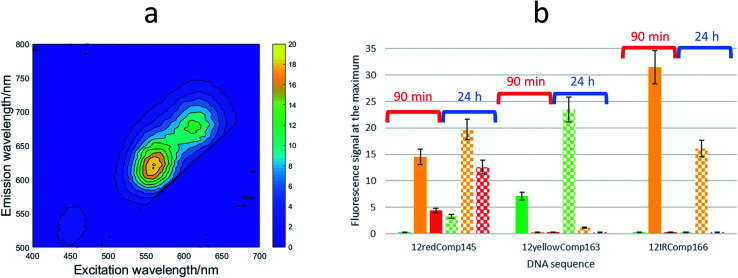
(a) EEM of 12redComp145-stabilized AgNCs after 24 hours in the dark at room temperature. (b) Dependence of fluorescence intensity of AgNCs stabilized by 12redComp145, 12yellowComp163 and 12IRComp166 with aging period. Solid bars represent fluorescence signals of samples measured 90 minutes after preparation (grouped under the red whisker), whereas dotted bars represent fluorescence signals of samples kept for 24 hours in the dark at room temperature (grouped under the blue whisker). Colours represent the following emission intervals: green (501–565 nm), orange (591–625 nm) and red (626–740 nm). In all cases, the conditions were DNA : Ag(i) : borohydride ratio of 1 : 6 : 6, 15 °C, 5 mM phosphate buffer pH 7, 2 μM DNA.

First, the effect on the fluorescence signal of the aging period at which DNA-stabilized AgNCs were aged was studied. To do this, AgNCs stabilized by 12redComp145, 12yellowComp163 or 12IRComp166 were synthesized at a ratio of 1 : 6 : 6 DNA : Ag(i):borohydride. For each of these DNA sequences, two samples were prepared. For one of them, the excitation-emission map (EEMs) was measured 90 minutes after preparation. The other sample was kept in the dark at room temperature for 24 h before doing a similar measurement (Fig. 5a). It was observed that the behaviours of the three sequences were clearly different (Fig. 5b). Whereas the position of the fluorescence bands of 12yellowComp163 and 12IRComp166 did not change after 24 h, the EEM of 12redComp145 showed the appearance of a new band in the green region, as well as an enhancement in the orange and red emissions. Also, the green emission of AgNCs stabilized by 12yellowComp163 increased over time, as was observed recently with AgNCs stabilized by RNA nanorings.^8^ In contrast, the orange emission of 12IRComp166 decreased after 24 h.

**Fig. 6:**
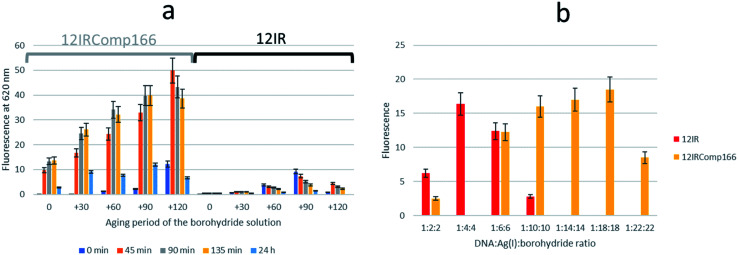
(a) Dependence of fluorescence intensity of AgNCs stabilized by 12IRComp16 (grouped under grey whisker) and 12IR (grouped under black whisker) on the aging period of borohydride solution and on the aging period of the synthesized AgNCs. Each series of bars (0, +30, +60, +90, +120) represents the aging period of the 200 μM borohydride solution (in minutes), whereas each colour represents the time at which fluorescence was measured after synthesis of respective AgNCs. The DNA : Ag(i):borohydride ratio was 1 : 10 : 10. (b) Dependence of fluorescence intensity of AgNCs stabilized by 12IR and IRComp166 on the DNA : Ag(i):borohydride ratio. The experimental conditions were excitation wavelength 560 nm, 15 °C, 5 mM phosphate buffer pH 7, 2 μM DNA.

Next, the need for the “fresh” condition of the borohydride solution was studied. The following experimental procedure was used. A 200 μM borohydride solution was prepared in ultrapure water. At time 0 minutes, the appropriate volume of this solution was added to a 12IRComp166 : Ag(i) (1 : 10) mixture in 5 mM phosphate buffer (pH 7) at room temperature. The final volume and ratio of the DNA : Ag(i):borohydride mixture were 250 μL and 1 : 10 : 10, respectively. Fluorescence emission spectra of this mixture were measured 0, 45, 90, 135 and 180 minutes after the addition of borohydride. Next, 30, 60 and 90 minutes after the preparation of the borohydride solution, additional 12IRComp166:Ag:borohydride (1 : 10 : 10) mixtures were prepared, and fluorescence spectra were also measured at the same time intervals as for the first mixture (Fig. 6a). The results showed strong fluorescence that depended strongly on two main factors: not only on the aging period of the AgNCs formed after the addition of borohydride to the 12IRComp : Ag(i) mixture, but also on the aging period of the 200 μM borohydride solution. The reason behind the need for a short aging period of the borohydride solution could be related to an optimal ratio between BH_4_^−^ (reactant) and BO_2_^−^ (product of the reaction with water), as previously reported in ethanol.^45^ Under those conditions, the BO_2_^−^ species could prevent the formation of large, non-fluorescent silver nanoparticles by aggregation of AgNCs, as observed in the formation and immobilization of AgPd nanoparticles.^46^ For comparison, a parallel experiment was done with 12IR, which is not able to fold intermolecularly or intramolecularly. As expected, the measured fluorescence was lower than in the case of the longer sequence. Even though fluorescence was enhanced after the aging period of borohydride solution, the fluorescence of the prepared AgNCs quickly decayed after preparation. This disagrees with the enhancement of AgNCs stabilized by 12IRComp166, which is probably related to the folding of this last sequence. Overall, it was concluded that the best conditions for measurement were achieved when using borohydride and AgNCs with 90- and 90 minute aging periods, respectively.

The influence of the DNA : Ag(i):borohydride ratio on the fluorescence intensity of the synthesized AgNCs was also studied for both short and long sequences. Fig. 6b shows the observed variation for AgNCs stabilized by 12IR or 12IRComp166. Similar results were obtained for the other sequences (Fig. S8†). For 12IR, the maximum intensity was obtained around the 1 : 4 : 4 ratio, which is similar to the DNA : Ag(i) ratio determined from CD-monitored titrations. In the same way, the maximum intensity was obtained close to the 1 : 18 : 18 ratio for the longer sequence.

Fig. S9† shows the EEM of short and long DNA-stabilized AgNCs at 1 : 6 : 6 and 1 : 22 : 22 (DNA : Ag(i) : borohydride) ratios. In the case of 12red and 12redComp145, the positions of the excitation and emission bands were similar, which suggested that the elongation of the strand did not affect the fluorescence of the 12red moiety. In the case of 12IR and 12IRComp166, a completely different situation was observed. Whereas 12IR emits near the IR region, 12IRComp166 emits in the orange region. This fact suggested that the elongation of the strand strongly affected the fluorescence of 12IR, probably because of the formation of secondary structures. Finally, despite the presence of minor fluorescence bands, 12yellow and 12yellowComp163 showed similar emission in the green region.

**Fig. 7:**
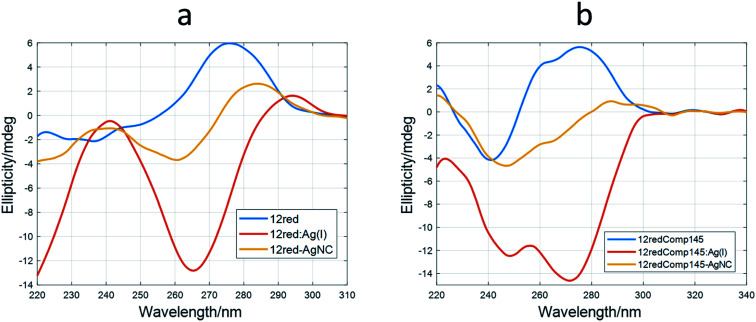
Experimental CD spectra of DNA (blue), DNA : Ag(i) complex (red) and DNA-stabilized AgNCs (yellow). (a) 12red, (b) 12redComp145. The experimental conditions were 15 °C, pH 7.2, 2 μM DNA. The ratio for AgNC synthesis was 1 : 6 : 6 (DNA : Ag(i) : borohydride).

Therefore, it was deduced that the storage period of AgNCs after their preparation was important to their fluorescent properties. Also, the EEM seems to depend strongly on the DNA sequence and, in consequence, on the secondary structure adopted by the DNA. This hypothesis implies that DNA would adopt a structure within the AgNCs like that observed in the bare DNA. To gain insight into this fact, CD spectra of the AgNCs formed by several sequences (DNA : Ag(i) : borohydride 1 : 10 : 10) were measured and compared with the respective Ag(i) : DNA complexes (Fig. 7).

### CD spectroscopy

It was observed that the shape of the CD spectrum of 12red-AgNCs (Fig. 7a) was intermediate between the spectrum of 12red and that previously recorded for the 12red:Ag(i) complex (Fig. 2a). Other authors have also observed this fact,^2,47^ which was explained in terms of different structural changes induced by CD in DNA by Ag than those induced by Ag(i). This was somewhat surprising as it would be expected that the reduction would not affect the overall structure of the Ag(i) : DNA complex, which is supposed to be stabilized by C·Ag(i)·C triplets. In this sense, Swasey *et al.* observed that CD spectra of DNA-stabilized AgNCs were like those recorded for DNA : Ag(i) complexes. However, in their case, the concentration of borohydride was half that of Ag(i).^48^

Similar behavior was observed for the long sequences (Fig. 5b and S10†). The CD spectrum of 12yellowComp163-AgNC is the most like that of free, unbound DNA. Our hypothesis is that reduction of Ag(i) by borohydride produces the removal of most of the base pairs stabilized by Ag(i), yielding a cluster^49^ or planar^50^ structure of silver species at the 5′ end rather than a rod involving the whole strand.^51^ It is interesting to note that AgNCs stabilized by 12yellowComp163 showed a CD spectrum most like that of bare DNA. This could be related to the fact that this sequence is the one that does not fold into a hairpin or intermolecular duplex.

Under the experimental conditions used in this study (2 μM DNA), no clear signal of induced CD was observed around 400 nm. As in the case of the oxidized complex, hardly any changes were observed upon heating of the AgNC solution, a fact which points to a very stable structure (data not shown).

### SE-HPLC

**Fig. 8:**
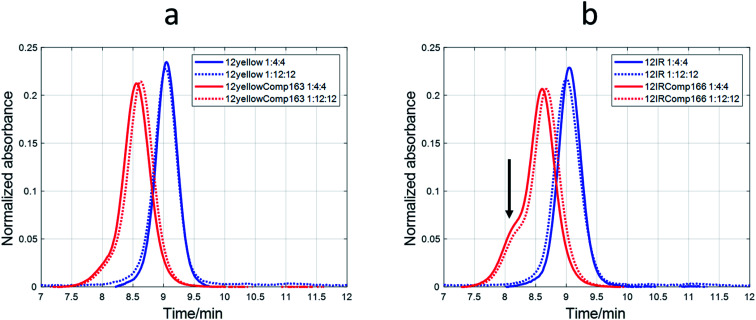
SE-HPLC chromatograms of AgNCs, which were synthesized at 1 : 4 : 4 and 1 : 12 : 12 DNA : Ag(i) : borohydride ratios and using four different DNA sequences. (a) 12yellow and 12yellowComp163. (b) 12IR and 12IRComp166. Experimental conditions were 5 μM DNA, 5 mM phosphate buffer, pH 7.1.

The nature of the DNA-stabilized AgNCs was also studied by means of SE-HPLC.^44,52,53^ For 12yellow and 12IR (Fig. 8) and 12red (Fig. S11†), a single elution band was observed despite the DNA : Ag(i) : borohydride ratio at which AgNCs were synthesized. This fact indicated the absence of mixtures of structures. For 12yellow-stabilized AgNCs with a 1 : 4 : 4 ratio, the retention time was 9.05 minutes. According to the calibration model, 12yellow (MW 3501.3 g mol^−1^) would elute at 9.08 minutes (as monomer) or 8.71 minutes (as dimer). Therefore, it was concluded that the elution band of 12yellow-stabilized AgNCs correspond to a DNA monomer. The small advance in elution would be due to the addition of Ag species, which produced an increase in the hydrodynamic volume of the DNA, resulting in a smaller retention time. A similar rationale may be applied to data from 12red or 12IR. Based on this hypothesis, these three short sequences would remain basically unfolded in the resulting AgNC, and an approximate value of the number of Ag species bonded to the sequence could be calculated from the difference between the calculated MW (from the *t*_R_*vs.* log(MW) calibration plot) and the known MW of each of the short DNA sequences. For 12red, 12yellow, and 12IR, the calculated number of Ag species bonded to each one was around 2 (1.9 ± 0.6, 1.6 ± 0.7 and 1.7 ± 0.6, respectively). In the case of the 1 : 12 : 12 ratio, the AgNCs stabilized by the short sequences eluted even earlier (8.98, 9.03 and 9.00 minutes for 12red, 12yellow and 12IR, respectively) than in the case of the 1 : 4:4 ratio. Assuming again that the DNA is an unfolded strand, the number of Ag species bonded to each DNA was 6.7 ± 0.7, 2.9 ± 0.7 and 5.1 ± 0.7, respectively.

A different behavior was observed in the case of probe sequences. Both 12redComp145 and 12yellowComp163 eluted as a single band despite the DNA : Ag(i) : borohydride ratio at which AgNCs were synthesized (Fig. 8a and S11†). For 12yellowComp163 and 1 : 4 : 4 ratio, the retention time was 8.55 minutes, whereas the calculated retention times for monomer and self-duplex were 8.48 and 8.12 minutes, respectively. First, it was observed that, for the same ratio, long sequences eluted before the shorter ones. This was the expected trend as they are larger molecules, have a greater hydrodynamic volume, and therefore do not enter the pores of the smaller filler, shortening the time they spend in the column. Secondly, unlike short sequences, the elution bands of long sequences appeared at retention times greater than those calculated for monomer species. Moreover, the retention times increased concomitantly with the ratio at which AgNCs were synthesized. The straightforward explanation was to consider that the presence of Ag species promoted some intramolecular folding, producing a reduction in the hydrodynamic volume. Finally, and due to the clear changes in the hydrodynamic volumes of long oligonucleotides, it was not possible to apply the calibration model based on the hypothesis of unfolded strands. Hence, it was not possible to determine the number of Ag species bound to each oligonucleotide.

In the case of 12IRComp166, a second band appeared around 8.1 minutes (Fig. 8b). According to the calibration plot, this band would correspond to the self-duplex (8.15 minutes), in agreement with *in silico* calculations and melting experiments.

### Hybridization properties of AgNCs with DNA and RNA sequences.

#### CD and stability studies

**Fig. 9:**
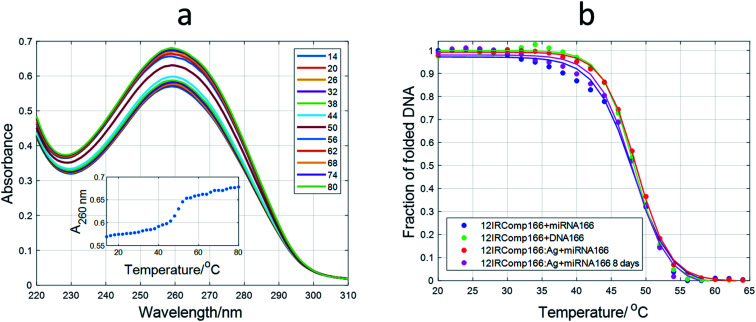
Melting experiments involving 12IRComp166. (a) Absorbance spectra recorded during the melting of the 12IRComp166 : DNA166 1 : 1 mixture. Inset: absorbance at 260 nm *vs. T*. (b) Plot of fraction of folded DNA *vs.* T for several mixtures. For each sequence, strand concentration was 1 μM, 5 mM phosphate buffer, pH 7.1.

Once the fluorescence of the AgNCs formed by 12redComp145, 12yellowComp163 and 12IRComp166 had been studied, the influence of the corresponding complementary DNA sequences on the analytical signal was investigated. First, the formation of the duplex structures was assessed by means of molecular absorption-monitored melting experiments. As a reference, the melting of the 1 : 1 mixture of 12IRComp166 and DNA166 was undertaken first (Fig. 9a). Multivariate analysis showed that the data could be well explained in terms of a cooperative, two-step unfolding with *T*_m_ equal to 50.7 °C and around 20% hyperchromicity at 260 nm, which suggests the unfolding of a duplex structure. The melting of a 1 : 1 mixture of 12IRComp166 and miRNA166 showed a slightly lower *T*_m_ value (48.4 °C) than the melting of the entirely DNA mixture. The lower *T*_m_ value of this DNA·RNA hybrid maybe explained by the relatively high purine content in the DNA strand, as demonstrated from both experimental and theoretical calculations.^54^ Next, the melting of a 1 : 1 mixture of miRNA166 and 12IRComp166 : Ag(i) (1 : 6) produced again a two-state melting transition with a *T*_m_ value of 48.5 °C. In contrast, the melting of a 1 : 1 mixture of miRNA166 and 12IRComp166 : Ag(i) (1 : 22) did not show any unfolding transition (Fig. S12†). Therefore, it was concluded that Ag species present in the 12IRComp166 : Ag(i) complex at ratio of 1 : 6 did not have any influence on the formation of the duplex structure. This fact implies that these Ag species are probably bound to the 5′ end (12red moiety). At a ratio of 1 : 22, additional bases in 12IRComp166 outside the 12red moiety are bonded to Ag species, hindering the formation of the duplex structure.

Finally, a 1 : 1 mixture of miRNA166 and 12IRComp166 : Ag(i) : borohydride (1 : 6 : 6) was prepared by the addition of an equimolar amount of miRNA166 to 24 hour aged AgNCs. The melting of this mixture showed a similar unfolding process to the previous experiments (*T*_m_ 48.8 °C and hyperchromicity around 18% at 260 nm). This result points to the maintenance of the Watson–Crick duplex structure.

#### Fluorescence studies

**Fig. 10:**
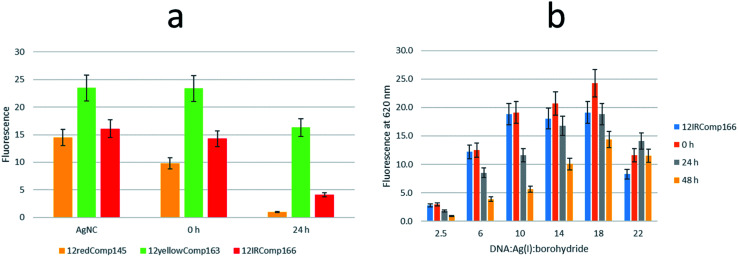
(a) Variation in the fluorescence signal of AgNCs stabilized by 12redComp145 (orange bars), 12yellowComp163 (green bars) and 12IRComp166 (red bars) after the addition of the complementary DNA sequences (1 : 1 ratio). In all cases, the DNA : Ag(i) : borohydride ratio was 1 : 6 : 6. The ‘AgNC’ series shows the initial fluorescence of DNA-stabilized AgNCs. The ‘0 h’ series shows the fluorescence signal just after the addition of the complementary DNA sequence, whereas the ‘24 h’ series shows the fluorescence at 4 °C 24 h after the addition of the complementary DNA sequence. (b) Variation in the fluorescence signal of AgNCs stabilized by 12IRComp166 after the addition of the complementary DNA166 sequence. Each series represents the variation in fluorescence for AgNCs prepared using different DNA : Ag(i) : borohydride ratios. Blue bars show the fluorescence of the respective 12IRComp166-stabilized AgNCs. Orange bars show the fluorescence just after the addition of complementary DNA166 (1 : 1 ratio), whereas grey and yellow bars show the fluorescence signal after 24 and 48 h, respectively. In all cases, experiments were carried out under the conditions of 5 mM phosphate buffer pH 7, 2 μM DNA, 15 °C.

The addition of complementary DNA sequences to AgNCs stabilized by 12redComp145, 12yellowComp163 and 12IRComp166 produced changes in fluorescence that depended on the nature of the stabilizing DNA (Fig. 10a). Immediately after the addition of the complementary DNA sequence, the fluorescence of the AgNCs formed by 12redComp145 and 12IRComp166 showed a small decrease. In contrast, the fluorescence of AgNCs stabilized by 12yellowComp163 did not show any immediate decrease in fluorescence. After 24 h, the decrease was clearer in the case of AgNCs stabilized by 12redCom1p45. This “turn-off” mechanism is similar to that previously reported^21^ but differs from the “turn-on” strategy described by Petty *et al.*^12^

The influence on the fluorescence of the resulting 1 : 1 mixture with DNA166 of the DNA : Ag(i) : borohydride ratio at which 12IRComp166-stabilized AgNCs were synthesized was studied (Fig. 10b). Upon addition of the DNA, small changes were observed at all ratios. A clear decrease was observed after 24 or 48 h. In the case of the 1 : 22 : 22 ratio, no decrease in fluorescence intensity was observed, which again pointed to the non-formation of the duplex structure.

**Fig. 11:**
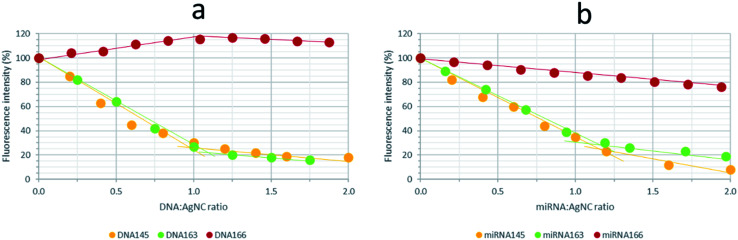
Variation in fluorescence intensity of the synthesized AgNCs with the addition of complementary DNAs (a) and miRNAs (b). AgNCs stabilized by 12redComp145, 12yellowComp163 or 12IRComp166 were synthesized under the conditions of 1 : 6 : 6 DNA : Ag(i) : borohydride ratio, 15 °C, 5 mM phosphate buffer pH 7, 2 μM DNA.

Finally, titrations of AgNCs stabilized by 12redComp145, 12yellowComp145 or 12IRComp166 (1 : 6 : 6) ratio, and which had been aged previously for 24 h at 4 °C, were titrated with their complementary DNAs or miRNAs (Fig. 11 and S13†). For 12redComp145 and 12yellowComp163, the fluorescence of AgNCs decreased clearly in the presence of increasing amounts of the complementary DNA, the observed variation being smaller beyond the 1 : 1 stoichiometry. As similar trends were observed in the presence of complementary miRNAs, this variation in the fluorescence signal could potentially be used to develop analytical methods for miRNA determination.

In the case of 12IRComp166, however, the addition of complementary DNA166 or miRNA166 did not produce any clear reduction in fluorescence under these experimental conditions. As the formation of AgNC-12IRComp166:DNA166 or AgNC-12IRComp166:miRNA166 duplexes had been demonstrated previously from the melting experiments, this odd variation in the fluorescence could be related to the kinetics of the duplex formation. The slow rate of this reaction could probably be related to the existence of a certain proportion of self-duplex 12IRComp166.

## Discussion

Recent years have witnessed an intense search for stable and highly fluorescent emitters that could be used for the efficient detection of biomolecules, such as miRNAs.^55^ In pioneering work, Richards *et al.*^25^ selected five 12-nucleotide long DNAs that were very efficient in templating the formation of highly fluorescent AgNCs. Each of these DNAs was able to generate AgNCs with specific fluorescent emissions ranging from 485 to 705 nm. The selected sequences had a high cytosine-rich sequence near the 3′ and 5′-ends with variable amounts of adenine and thymine bases. Yang *et al.*^21^ described that linking one of these selected DNAs (12red, Table 1) to a DNA sequence complementary to a microRNA did not disrupt the ability of 12red to template the formation of highly fluorescent AgNCs. However, upon hybridization with the target miRNA, the capacity for templating the formation of AgNCs became less efficient, resulting in a “turn-off” strategy that could be used to determine miRNA content in real samples. From the results described in that work, we aimed to develop an analytical method for the simultaneous analysis of several miRNAS based on fluorescence measurements and multivariate calibration. Prior to this, we studied the fundamental aspects of AgNC synthesis and the key variables that affect the analytical signal.

First, we characterized the short (12red, 12yellow, 12IR) and long (12redcomp145, 12yellowcomp163, 12IRcomp166) DNA sequences by CD, demonstrating that the short oligonucleotides are low structured but the longer sequences form either intramolecular duplexes (hairpins) or intermolecular duplexes. Then binding of Ag(i) cations to these sequences was measured by CD, allowing the determination of the number of Ag(i) species bound to each DNA. Short sequences bind up to 5–6 ions per DNA molecule, but the longer DNA probes presented a more complex scenario, binding up to 22–25 Ag(i) species. CD spectra of the DNA : Ag(i) complexes show spectral features that resemble those present in CD spectra of i-motif structures. Hence, several authors have proposed the presence of C–Ag(i)–C triads ^3536^. Multivariate analysis based in soft modelling of the whole set of spectra recorded during titration of the long DNA sequences with Ag(i) indicated the presence of an intermediate complex with DNA : Ag(i) stoichiometry of approximately 1 : 10. The next step was the synthesis of DNA-stabilized AgNCs and a study of the influence of some variables on the fluorescence of the formed AgNCs. In our opinion, many studies dealing with the characteristics and uses of AgNCs do not reflect enough on the critical importance of these variables on the robustness of AgNC synthesis, and on the analytical methods based on their use. Finally, the binding of the DNA-stabilized AgNCs to complementary DNAs and miRNAs was studied. The study, which is planned to be extended to the simultaneous determination of several miRNAs, was limited here to a qualitative description on the variation in the fluorescence of DNA-stabilized AgNCs upon hybridization with miRNAs. It was observed that 12IRComp166-stabilized AgNCs did not produce the same variation as the other two DNA sequences. This fact was explained by the self-duplex formation of this DNA sequence, which was not observed in the case of 12redComp145 or 12yellowComp163.

The variables that govern the fluorescence characteristics, mainly the position of the excitation/emission bands, are still a matter for discussion, despite the applications of AgNCs in several fields. In their early work, Ritchie *et al.* found that no stoichiometries, and hence cluster sizes, were favored in the synthesis of AgNCs stabilized by a dC_12_ sequence. The observed blue/green- and red-emitting species were explained as a result of a redox reaction, being unrelated to different AgNCs.^27^ This observation seems to be in disagreement with another study, where it was suggested that the fluorescence of DNA-stabilized AgNCs depends on the number of Ag atoms.^56^ These authors used DNA hairpins with 3 to 12 cytosines in the loop and they were able to observe that the size of the loop tunes the fluorescent properties in the AgNCs analyzed by mass spectrometry. By using MS-HPLC, Schultz and Gwinn determined the number of DNA strands and Ag species present in a palette of AgNCs emitting from green to IR.^57^ They concluded that the number of Ag species is not a key variable to explain the origin of the color, but probably the Ag-nucleic base interactions. Interestingly, these authors determined that 10–12 Ag species are present in AgNCs stabilized by a single DNA strand, whereas dimeric structures accommodate around 21 Ag species. In a recent study, 1 : 15 and 1 : 16 DNA : Ag(i) stoichiometries were proposed for single stranded DNAs,^58^ whereas a 1 : 16 stoichiometry was observed for two DNA decamers that take on a horse-shoe-like conformation.^59^ Another interesting head-to-head binding of two DNA hairpins, bridged by a silver nanocluster, resulting in the modelling of a dimeric structure harboring an Ag_12_ cluster.^53^ Overall, these values agree well with the stoichiometries determined in our work for the long sequences, suggesting that the 1 : 25 complex depicted in Fig. 4a–c would correspond to a dimer. This fact would explain why the AgNCs synthesized at a 1 : 22 : 22 ratio did not form a duplex structure with the complementary miRNA. On the other hand, the role of the spacer length and nature of the bases within it were studied systematically.^60^ It was concluded that fluorescence was clearly enhanced when a length of three to five nucleotides, as well as adenosine and thymine, were present in the DNA-stabilizing sequence. In our case, 12yellow, which shows a spacer that fulfills this criterion, is the sequence that produces the more intense fluorescence when introduced into the corresponding AgNC (Fig. 10a). Finally, it has also been demonstrated that the presence of thermally stable secondary structures in DNA dramatically increases the intensity of red fluorescence.^13^

Other variables that influence the fluorescence characteristics of AgNCs are pH, especially in the case of cytosine-rich sequences as they could form i-motif structures at a pH below approximately 6.5.^27^ Other studies, however, have not observed this decrease in long cytosine-rich sequences.^61^ By using a DNA sequence similar to the ones used in this work, Shah *et al.* studied the influence on the fluorescence of the nature of added salts, solvents, buffers and even the counterions that accompany DNAs supplied by different laboratories.^62^ Overall, all these studies demonstrated that the experimental variables at which AgNCs are synthesized should be critically controlled and described.

## Conclusions

The use of the DNA-templated formation of extremely fluorescent AgNCs for the detection of three miRNAs has been explored. The results obtained during this study reinforced the importance of the choice of the experimental conditions under which the synthesis of AgNCs is undertaken, as well as the role of DNA secondary structures on their fluorescence properties. Hence, DNA sequences prone to form stable self-duplexes may not be suitable for the detection of miRNAs by means of this “turn-off” strategy. This fact should be taken into account when developing analytical methods for the detection of these analytes.

## Conflicts of interest

There are no conflicts to declare.

## Supplementary Material

RA-011-D1RA00194A-s001
