# Supplementary material for: Studies on the interactions of Ag(i) with DNA and their implication on the DNA-templated synthesis of silver nanoclusters and on the interaction with complementary DNA and RNA sequences
Source: RSC Adv. 2021 Mar 1;11(15):9029–42. doi: 10.1039/d1ra00194a (PMC8695332; doi:10.1039/d1ra00194a)

Electronic Supplementary Material (ESI) for RSC Advances.
This journal is © The Royal Society of Chemistry 2021

Supplementary Material

Studies on the interactions of Ag(I) with DNA and their implication on DNA-templated synthesis of silver nanoclusters and on the interaction with complementary DNA and RNA sequences

Alejandra de la Hoz[1], Alba Navarro[1], Anna Aviñó[2], Ramon Eritja[2], Raimundo Gargallo[1]*

1. Dept. of Chemical Engineering and Analytical Chemistry, University of Barcelona, Marti i Franquès 1, E-08028 Barcelona, Spain

2. Institute for Advanced Chemistry of Catalonia (IQAC-CSIC), CIBER-BBN, Jordi Girona 18-26, E-08034 Barcelona, Spain

Contents:

Figure S1. *In silico* prediction of folded structures.

Figure S2. CD spectra of 12red at pH 5.0 and 7.1

Figure S3. CD spectra recorded along the melting experiment of 12IRComp166.

Figure S4. Melting experiment of 12yellowComp163.

Figure S5. CD spectra recorded along the titration of 12yellow and 12IR with Ag(I).

Figure S6. CD spectra recorded along the titration of 12yellowComp163 and 12IRComp166 with Ag(I).

Figure S7. Comparison of the residual variance not explained by the MCR-ALS models of two or three species.

Figure S8. Dependence of fluorescence with DNA:Ag(I):borohydride ratio for 12red and 12redComp145, and 12yellow and 12yellowComp163.

Figure S9. EEM of short and long DNA-stabilized AgNCs.

Figure S10. CD spectra of 12yellowComp163 and 12IRComp166 AgNCs

Figure S11. SE-HPLC data.

Figure S12. Melting experiment of the 1:1 12IRComp166:Ag(I) (1:22) : miRNA166 mixture.

Figure S13. EEM of 12yellowComp163-stabilized AgNCs in the presence of increasing concentrations of miRNA163.

Figure S1. ***In silico*** **prediction of folded structures**

In silico calculations were done at 15°C and 20 mM Na^+ concentration by using the *mfold* and OligoAnalyzer version 3.1 methods.

DNA sequence	Proposed hairpin	Calculated Gibbs free energy for hairpin formation (kcal·mol^{-1})	Proposed self-duplex	Calculated Gibbs free energy for self-duplex formation (kcal·mol^{-1})
12red	-	-	-	-
12yellow	-	-	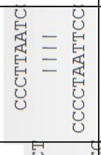	-4.8
12IR	-	-	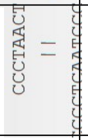	-1.0
12redComp145	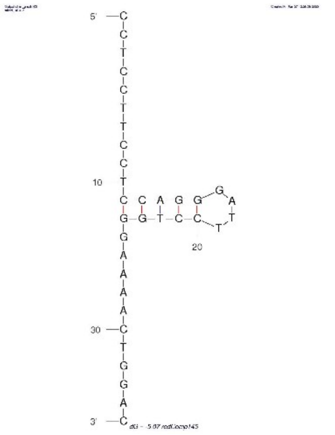	-5.4		-9.7
12yellowComp163	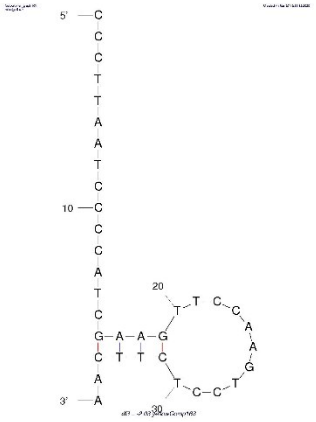	-2.3		-6.8

12IRComp166	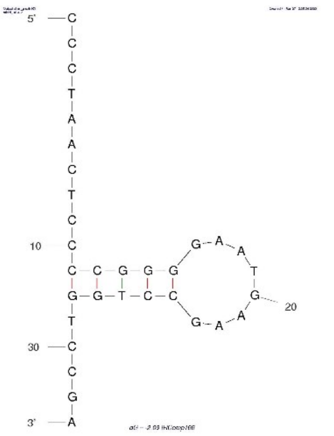	-2.4		-25.2

Calculated concentrations of unfolded strand, hairpin, and self-duplex at equilibrium

Sequence	$\Delta G_{hairpin}$ (kcal·mol^{-1})	$\Delta G_{self\text{-}duplex}$ (kcal·mol^{-1})	[unfolded] (M)	[hairpin] (M)	[self-duplex] (M)	$\Delta G_{probe:analyte\ duplex}$ (kcal·mol^{-1})
12red	-	-	-	-	-	-
12yellow	-	-4.8	*$4.8\cdot10^{-6}$*	-	$1.0\cdot10^{-7}$	-
12IR	-	-1.0	*$5.0\cdot10^{-6}$*	-	$1.4\cdot10^{-10}$	-
12redComp145	-5.4	-9.7	$4.0\cdot10^{-10}$	*$5.0\cdot10^{-6}$*	$3.6\cdot10^{-12}$	-45.4
12yellowComp163	-2.3	-6.8	$8.8\cdot10^{-8}$	*$4.9\cdot10^{-6}$*	$1.1\cdot10^{-9}$	-43.4
12IRComp166	-2.4	-25.2	$4.5\cdot10^{-13}$	$2.9\cdot10^{-11}$	*$2.5\cdot10^{-6}$*	-45.3

Calculated thermodynamic values at 15°C, 20 mM Na^{+} and 5 μM DNA concentration using the OligoAnalyzer version 3.1 [27]. Concentrations of unfolded strand, hairpin and self-duplex at equilibrium were calculated with the computer algebra system Maxima (v. 5.44.0). Values in italics emphasize the predominant species.

Figure S2. CD-monitored melting experiments of 12red at pH 5.0 and 7.1. Thermally induced variation of the CD spectra of 12red at pH 5.0 (a) and 7.1 (b). DNA concentration 2 μM, 20 mM acetate or phosphate buffer. Insets: variation of the ellipticity at 280 nm *vs*. T.

The i-motif structure is formed by cytosine-rich sequences at acidic pH and even neutral pH and low temperature. This structure consists on two parallel duplexes running in an antiparallel way, which are stabilized by the formation of C·H·C$^+$ triplets. Due to the requirement of protonation of one of the cytosine bases, the stability of i-motif structures is strongly pH-dependent. In the case of 12red, a melting experiment done at pH 7.0 revealed a small unfolding (Figure S2b) that was related to the minor formation of i-motif structure at this pH.

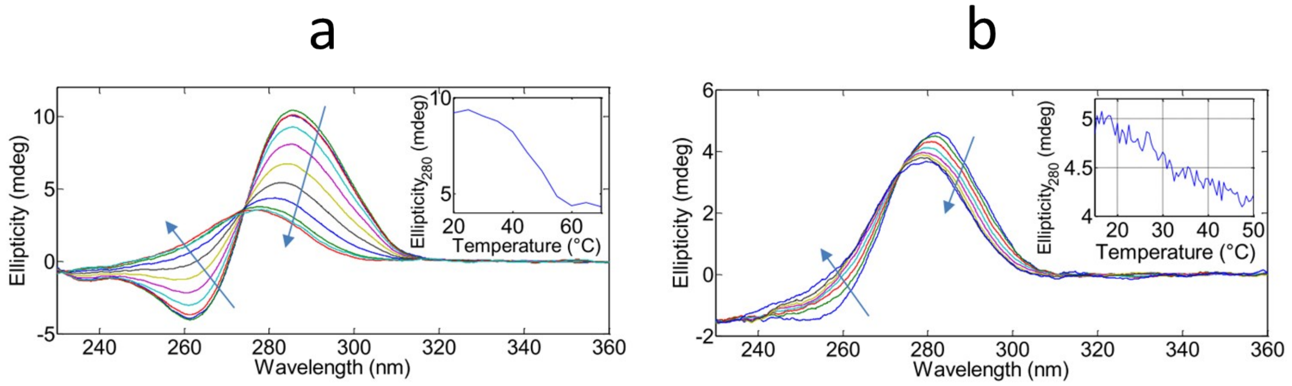

Figure S3. CD spectra recorded along the melting experiment of 12IRComp166. DNA concentration 2 μM, 5 mM phosphate buffer, pH 7.2.

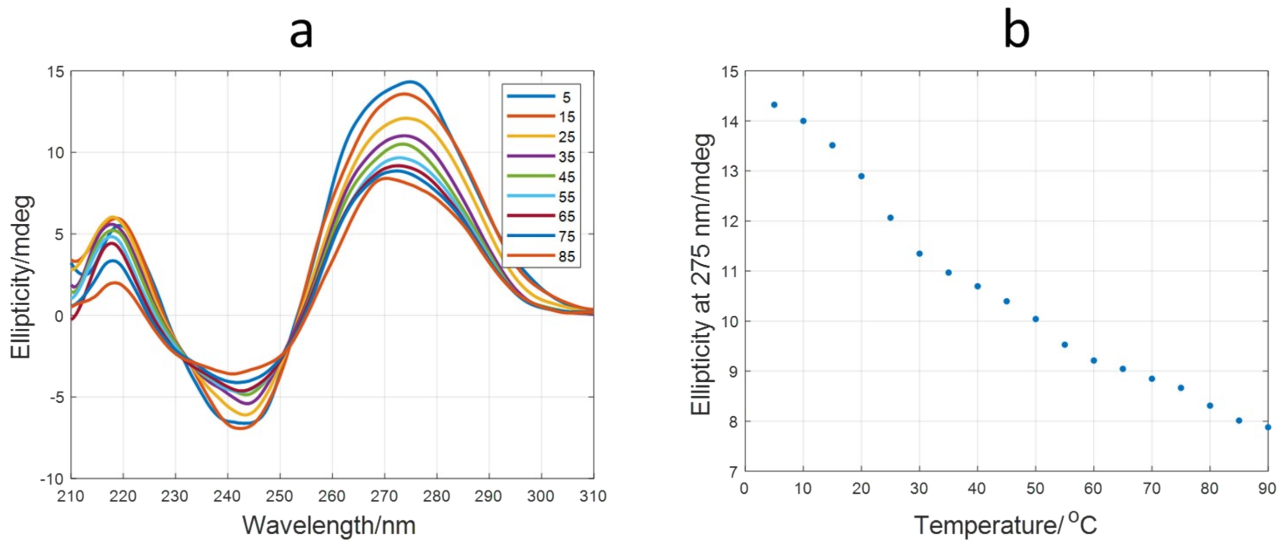

Figure S4. Melting experiment of 12yellowComp163. Absorbance spectra measured along the melting (a). Inset: absorbance at 295 nm vs. T, Calculated distribution diagram (b) and pure spectra (c). DNA concentration 2 μM, 5 mM phosphate buffer, pH 7.2.

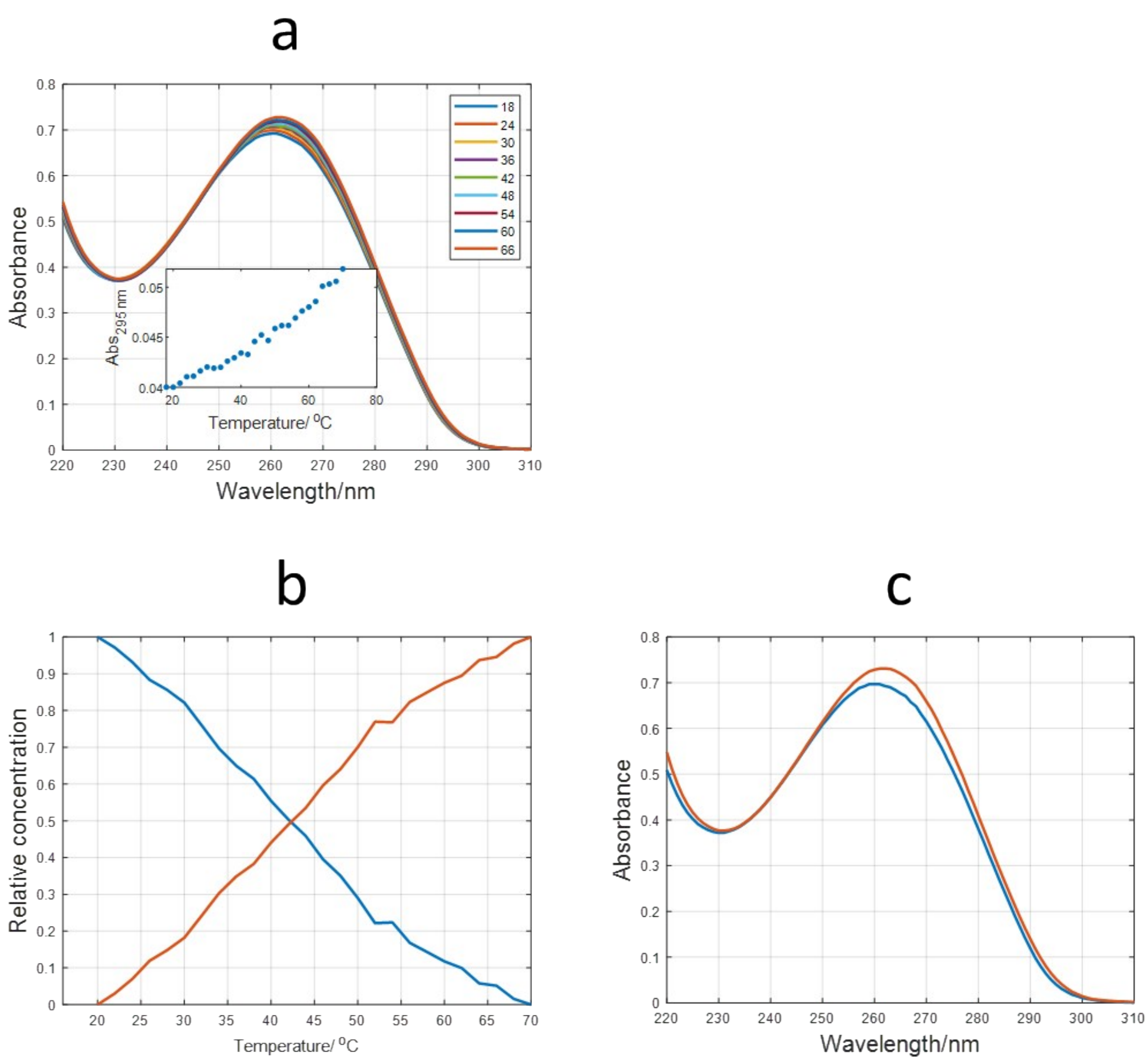

Figure S5. CD spectra recorded along the titration of 12yellow (a) and 12IR (b) with Ag(I). Legends show the Ag(I):DNA ratio at which CD spectra were measured. CD at 266 nm vs. Ag(I):DNA ratio for 12yellow (c) and 12IR (d). Experimental conditions were 5 mM phosphate buffer, pH 7.1, 15°C, DNA concentration was 2 μM.

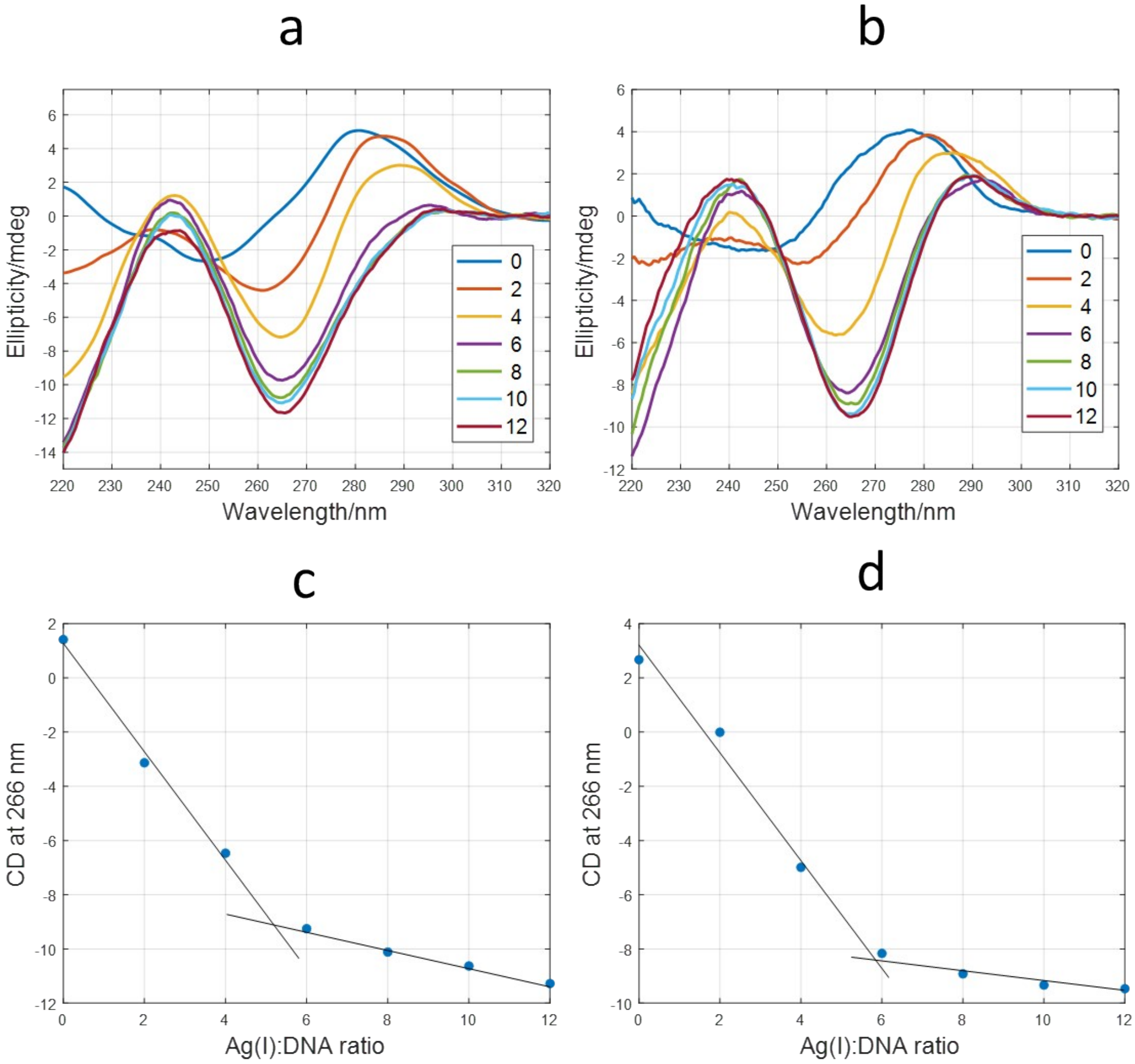

Figure S6. CD spectra recorded along the titration of 12yellowComp163 (a) and 12IRComp166 (b) with Ag(I). Legends show the Ag(I):DNA ratio at which CD spectra were measured. CD at 275 nm vs. Ag(I):DNA ratio for 12yellowComp163 (c) and 12IRComp166 (d). Experimental conditions were 5 mM phosphate buffer, pH 7.1, 15°C, DNA concentration was 2 μM.

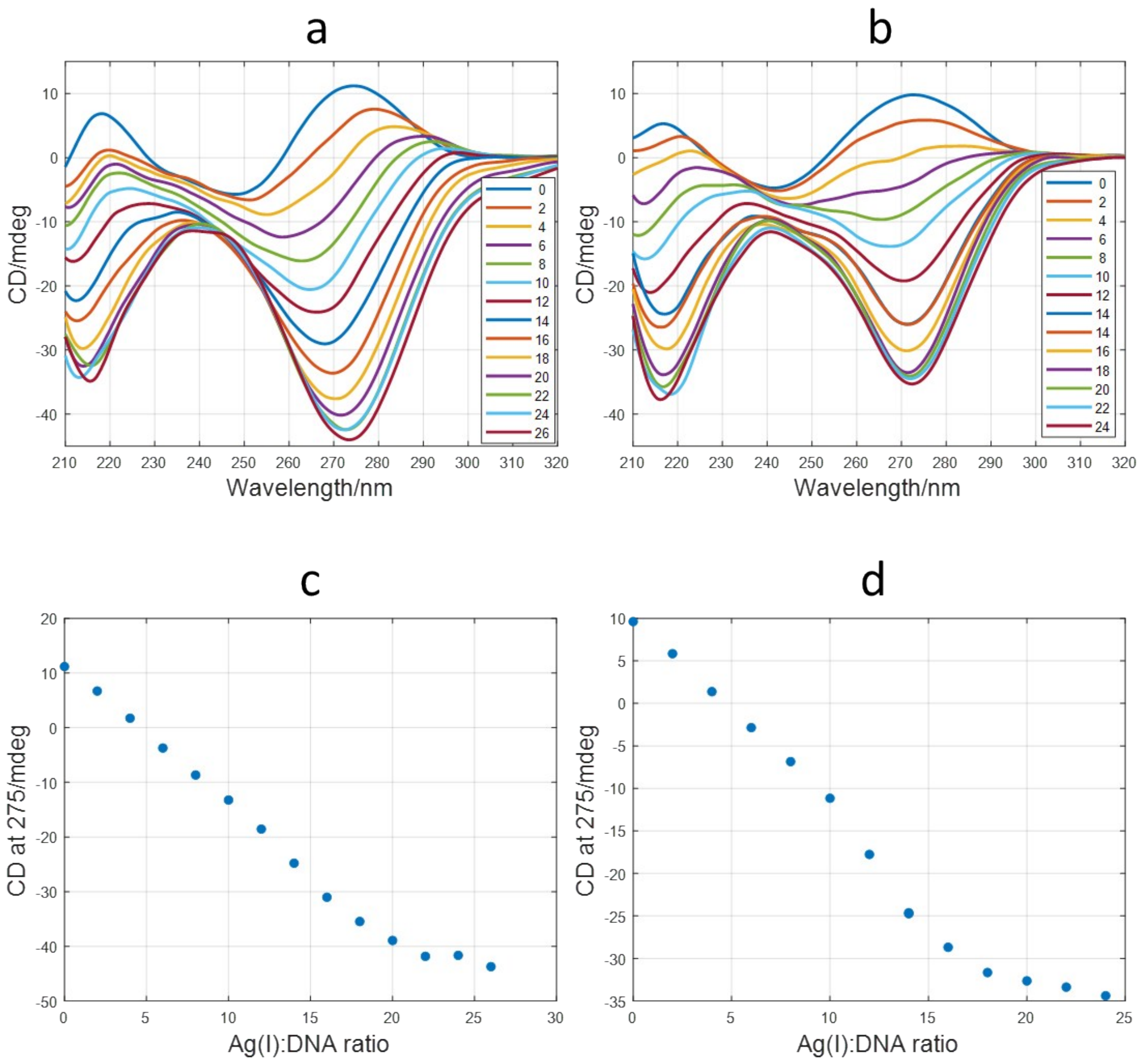

Figure S7. Comparison of the residual variance not explained by the MCR-ALS models of two or three species.

In all cases, the residual variance (matrix **E** in equation 2) were calculated according to the equation **E** = **D** – **C**·**S.**

A measure of the goodness of the fitting procedure is given by three magnitudes. First, the standard deviation of residuals in matrix **E** should be near that of the instrumental technique, which is around 0.3 millidegrees (mdeg). Second, the lack of fit is the percentage of the data in **D** that is not explained by the product of **C** and **S**. According to our experience, this value should be lower than 5%, approximately. Finally, the parameter R^2 is also related with the variance explained and should be near 1.

12redComp145 Considering 2 species Considering 3 species

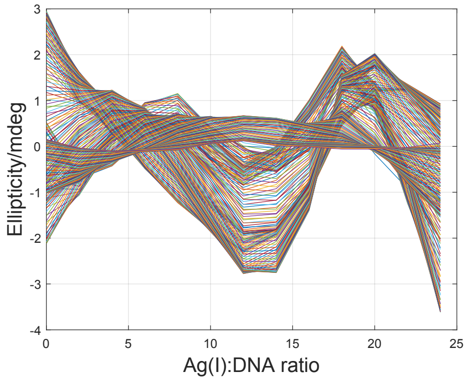

lack of fit: 9.1 %

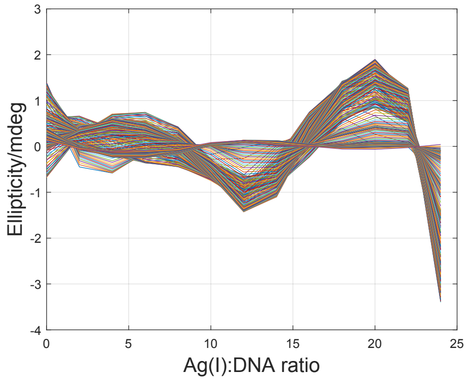

lack of fit: 3.8 %

12yellowComp163

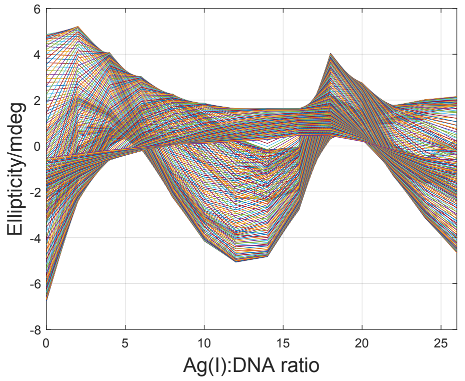

lack of fit: 12.3 %

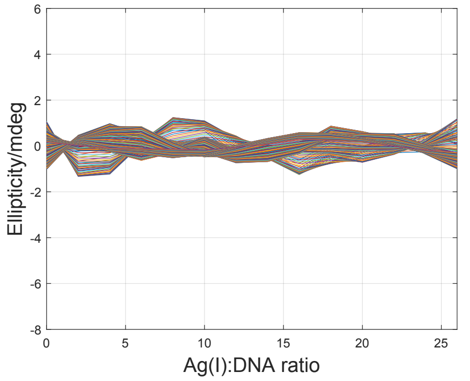

lack of fit: 2.8 %

12IRComp166

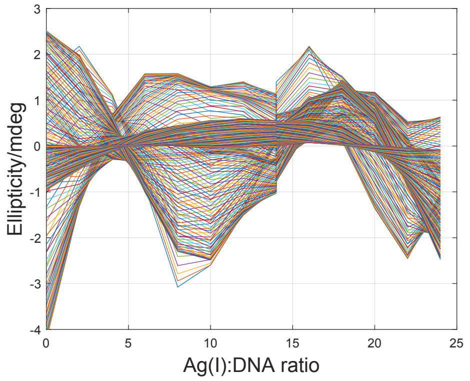

lack of fit: 6.7 %

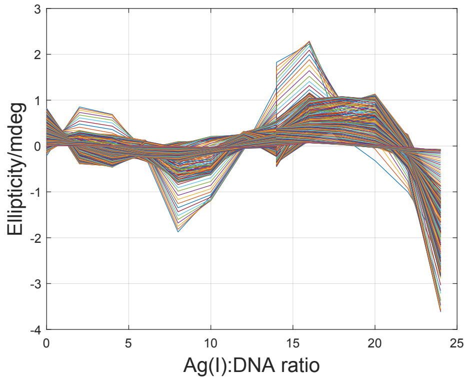

lack of fit: 4.0 %

DNA sequence	number of components (*nc*)	Standard deviation of residuals	lack of fit (%)	R^2
12redComp145	2	1.04	9.1	0.9917
	3	0.44	3.8	0.9985
12yellowComp163	2	2.01	12.3	0.9848
	3	0.45	2.8	0.9992
12IRComp166	2	0.95	6.7	0.9954
	3	0.57	4.0	0.9983

Figures of merit of multivariate analysis with MCR-ALS.

Figure S8. Dependence of fluorescence with DNA:Ag(I):borohydride ratio for 12red and 12redComp145 (a), and 12yellow and 12yellowComp163 (b). DNA concentration 2 μM, 5 mM phosphate buffer, pH 7.2.

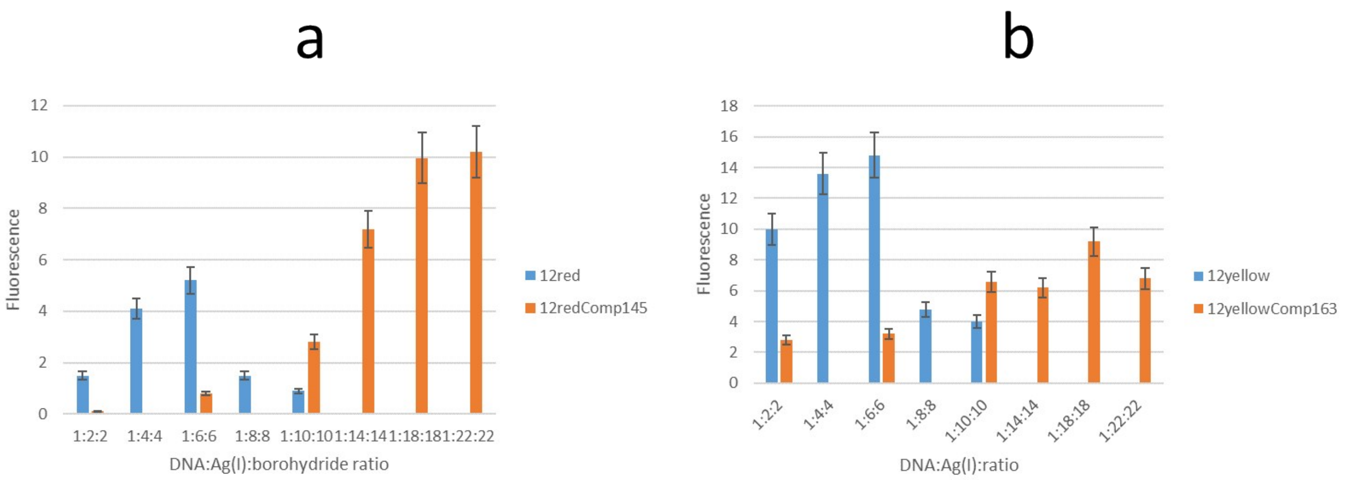

Figure S9. EEM of short and long DNA-stabilized AgNCs. 12red (a), 12IR (b), 12yellow (c), 12redComp145 (d), 12yellowComp163 (e) and 12IRComp166 (f). Short and long DNA-stabilized AgNCs were synthesized using a 1:6:6 and 1:22:22 DNA:Ag(I):borohydride ratio, respectively. DNA concentration was 5 μM, 5 mM phosphate buffer, pH 7.2, 15°C.

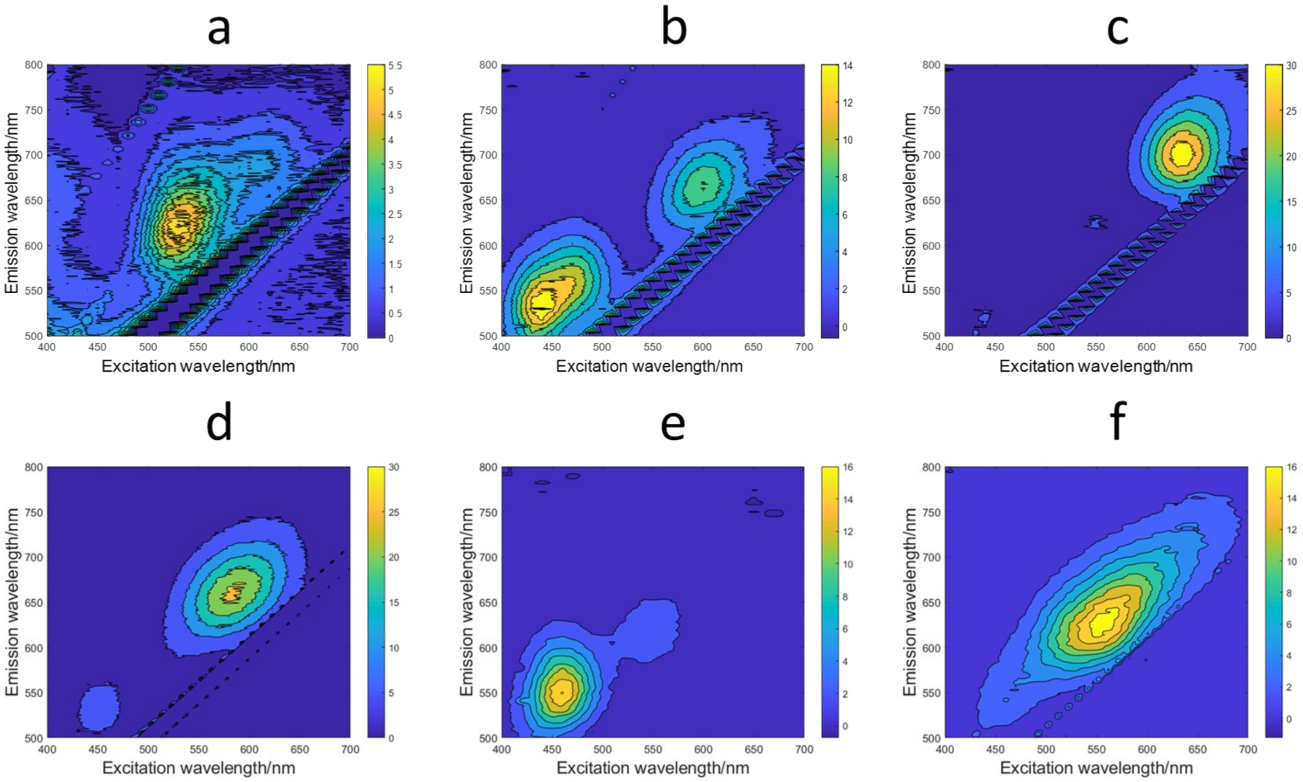

Figure S10. CD spectra of 12yellowComp163 and 12IRComp166 AgNCs. DNA (blue), DNA:Ag(I) complex (red) and DNA-stabilized AgNCs (black). (a) 12yellowComp163, (b) 12IRComp166. The experimental conditions were 15^oC, pH 7.2, 2 μM DNA. The ratio for AgNCs synthesis was 1:6:6 (DNA:Ag(I):borohydride).

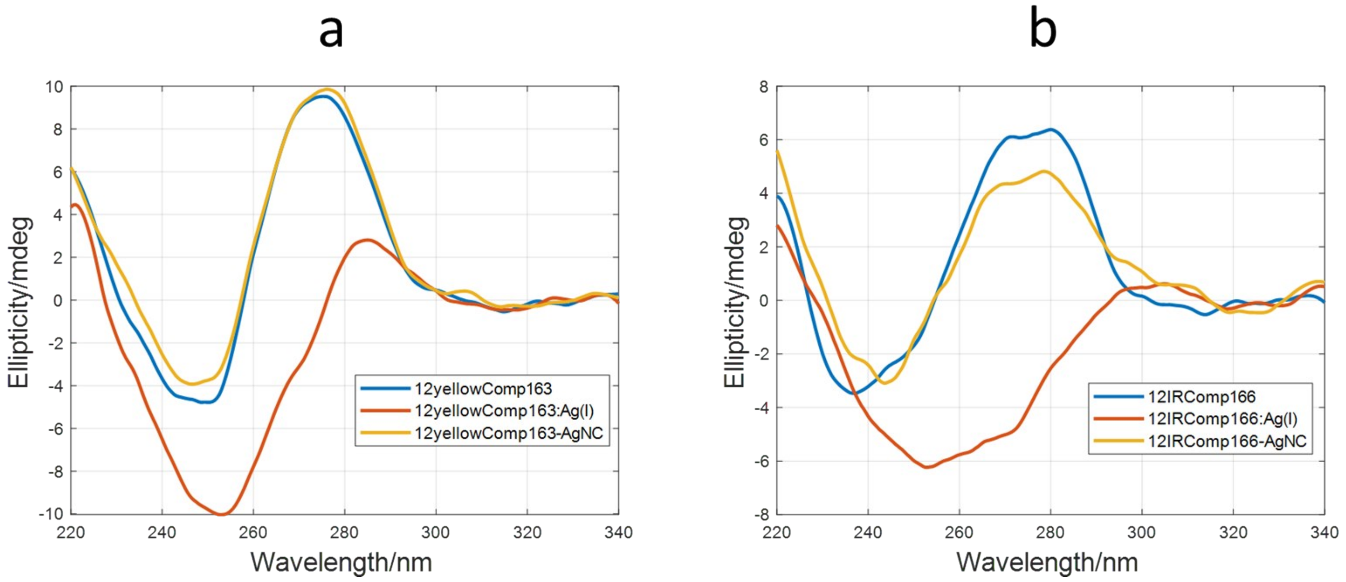

Figure S11. SE-HPLC data.

SE-HPLC is a technique where molecules are separated according to their hydrodynamic volume. In our case, this volume corresponds to that of the DNA (whether folded or unfolded), together with bonded Ag species, as well as with other counterions and water molecules in close vicinity. The hydrodynamic volume strongly depends on the structure of DNA, eluting folded monomeric structures later than the corresponding unfolded sequence. On the other hand, dimeric structures will elute earlier than the monomeric counterparts. The calibration model was built by plotting the retention time (t_R) of a set of T_x standards *vs.* their molecular weight (actually, $\log_{10}$(MW). This model allowed the calculation of the MW of any unfolded strand provided its experimentally measured t_R value.

DNA	MW	log(MW)	tR exp (min)
T15	4501	3.653	8.93
	4501	3.653	8.95
T20	6019.1	3.780	8.79
T25	7539.3	3.877	8.67
T30	9064	3.957	8.58
T45	13627	4.134	8.35
	13627	4.134	8.37

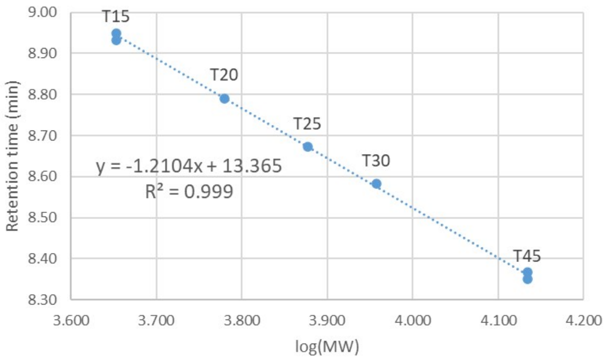

Samples

DNA	ratio	tR exp (min)	Nature	MW (g/mol)	log(MW)	tR calc (min)	Dif (%)	og MW cal	MW calc	Ag atoms
12red	1:4:4	9.05	Monomer	3468.3	3.540	9.08	-0.3%	3.56	3672.69	1.9
	1:4:4		Dimer	6936.6	3.841	8.72				
	1:12:12	8.98	Monomer	3468.3	3.540	9.08	-1.1%	3.62	4171.98	6.5
	1:12:12		Dimer	6936.6	3.841	8.72				
12yellow	1:4:4	9.05	Monomer	3501.3	3.544	9.08	-0.3%	3.56	3672.69	1.6
	1:4:4		Dimer	7002.6	3.845	8.71				
	1:12:12	9.03	Monomer	3501.3	3.544	9.08	-0.5%	3.58	3791.01	2.7
12IR	1:4:4	9.05	Monomer	3486.3	3.542	9.08	-0.3%	3.56	3672.69	1.7
	1:4:4		Dimer	6972.6	3.843	8.71				
	1:12:12	9.00	Monomer	3486.3	3.542	9.08	-0.9%	3.61	4039.21	5.1

DNA	ratio	tR exp (min)	Nature	MW (g/mol)	log(MW)	tR calc (min)	Dif (%)
12redComp145	1:4:4	8.62	Monomer	10667.9	4.028	8.49	1.5%
	1:12:12	8.65	Monomer	10667.9	4.028	8.49	
12yellowComp163	1:4:4	8.55	Monomer	10835.1	4.035	8.48	0.8%
	1:12:12	8.63	Monomer	10835.1	4.035	8.48	
12IRComp166	1:4:4	8.15	Dimer	20169.2	4.305	8.15	-0.1%
	1:4:4	8.60	Monomer	10084.6	4.004	8.52	
	1:12:12	8.15	Dimer	20169.2	4.305	8.15	-0.1%
	1:12:12	8.68	Monomer	10084.6	4.004	8.52	1.9%

Normalized SE-HPLC profiles of AgNCs stabilized by 12red or 12redComp145 at 1:4:4 and 1:12:12 (DNA:Ag(I):borohydride) ratio.

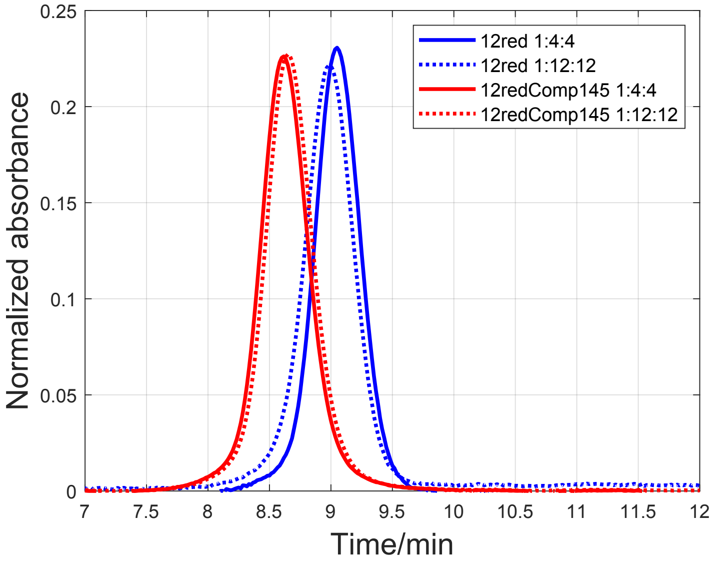

Figure S12. Melting experiment of the 1:1 12IRComp166:Ag(I) (1:22) : miRNA166 mixture. (a) Absorbance spectra recorded along the melting. (b, top) Absorbance at 260 nm *vs.* T. (b, bottom) First derivative of absorbance at 260 nm *vs.* T. Strand concentration was 1 μM, 5 mM phosphate buffer, pH 7.2.

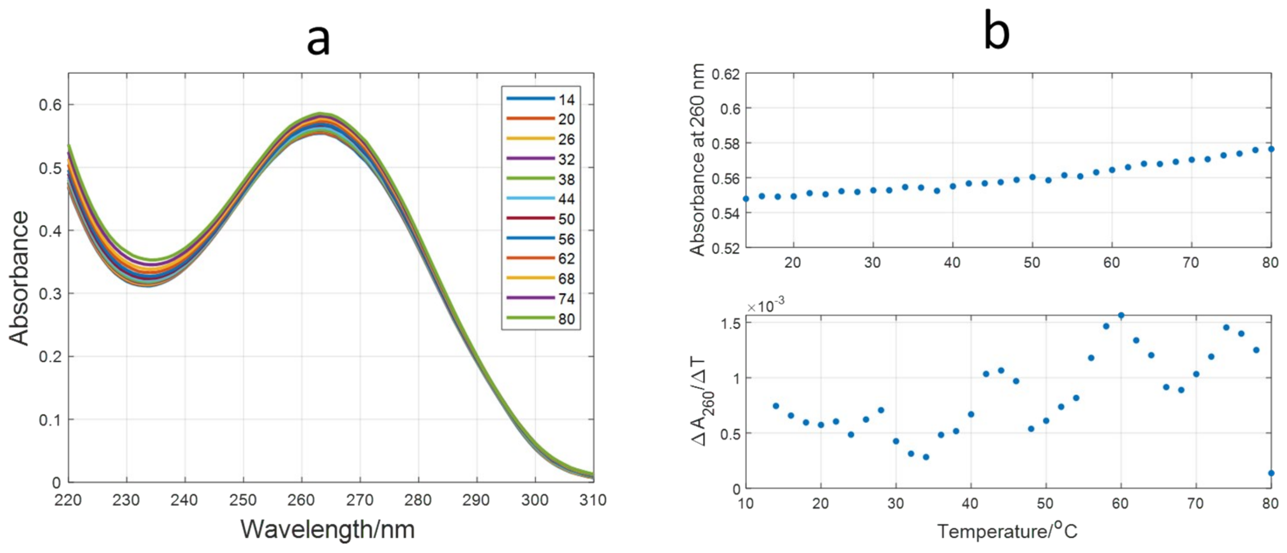

Figure S13. EEM of 12yellowComp163-stabilized AgNCs in the presence of increasing concentrations of miRNA163. (a) 0 microM miRNA163, (b) 4.3 microM miRNA163, (c) 9.3 microM miRNA163. Initial 12yellowComp163 concentration was 5 μM. AgNCs synthesized at 1:6:6 DNA:Ag(I):borohydride ratio. 5 mM phosphate buffer, pH 7.2, 15°C.

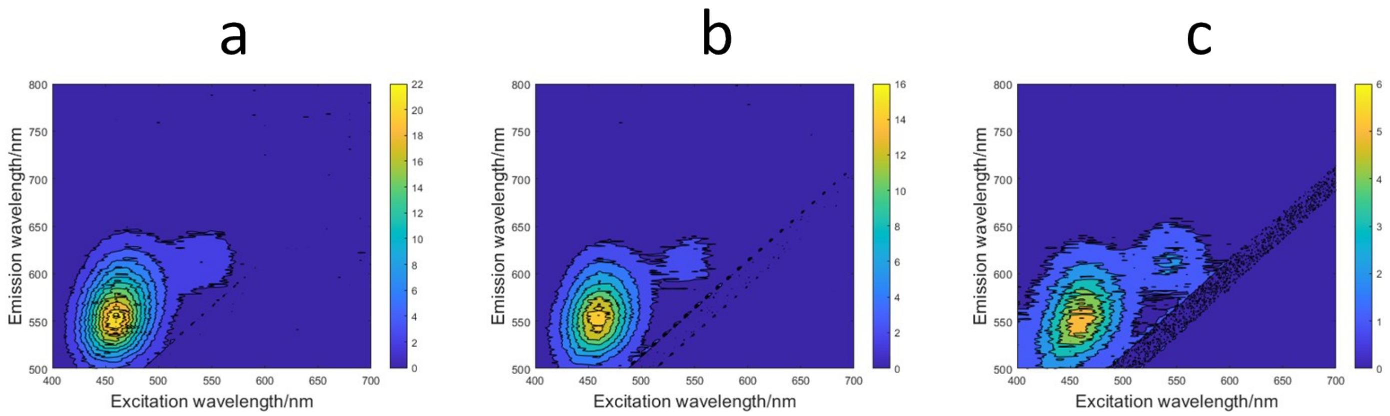

Supplement: RA-011-D1RA00194A-s001 [file RA-011-D1RA00194A-s001.pdf]
